# Tau Aggregation Inhibiting Peptides as Potential Therapeutics for Alzheimer Disease

**DOI:** 10.1007/s10571-022-01230-7

**Published:** 2022-05-21

**Authors:** Isabelle Aillaud, Susanne Aileen Funke

**Affiliations:** Institute of Bioanalysis, Coburg University of Applied Sciences, Coburg, Germany

**Keywords:** Tauopathies, Alzheimer disease, Tau, Aggregation inhibitors, Peptides

## Abstract

Alzheimer disease (AD) is the most common progressive neurodegenerative disorder. AD causes enormous personal and economic burden to society as currently only limited palliative therapeutic options are available. The pathological hallmarks of the disease are extracellular plaques, composed of fibrillar amyloid-β (Aβ), and neurofibrillary tangles inside neurons, composed of Tau protein. Until recently, the search for AD therapeutics was focussed more on the Aβ peptide and its pathology, but the results were unsatisfying. As an alternative, Tau might be a promising therapeutic target as its pathology is closely correlated to clinical symptoms. In addition, pathological Tau aggregation occurs in a large group of diseases, called Tauopathies, and in most of them Aβ aggregation does not play a role in disease pathogenesis. The formation of Tau aggregates is triggered by two hexapeptide motifs within Tau; PHF6* and PHF6. Both fragments are interesting targets for the development of Tau aggregation inhibitors (TAI). Peptides represent a unique class of pharmaceutical compounds and are reasonable alternatives to chemical substances or antibodies. They are attributed with high biological activity, valuable specificity and low toxicity, and often are developed as drug candidates to interrupt protein–protein interactions. The preparation of peptides is simple, controllable and the peptides can be easily modified. However, their application may also have disadvantages. Currently, a few peptide compounds acting as TAI are described in the literature, most of them developed by structure-based design or phage display. Here, we review the current state of research in this promising field of AD therapy development.

**Graphical Abstract:**

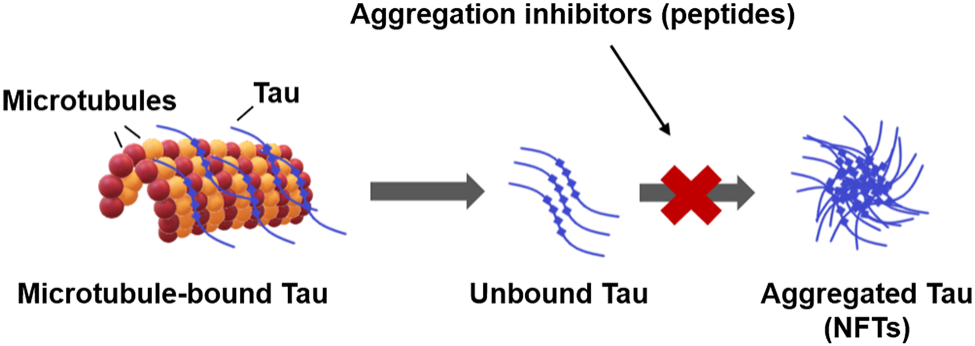

## Introduction

Tauopathies are a variety of progressive neurodegenerative disorders, characterized by the deposition of the abnormally aggregated microtubule-associated protein Tau. They include about 20 diseases, e.g. Huntington disease (HD), progressive supranuclear palsy (PSP), argyrophilic grain disease (AGD), primary age-related tauopathy (PART), chronic traumatic encephalopathy (CTE), and, most abundant, Alzheimer disease (AD) (Arendt et al. 2016).

Today, over 55 million people live with dementia worldwide, with forecasts reaching 78 million by 2030, and dementia is now the 7th leading cause of mortality globally (World Alzheimer Report 2021). AD is the most common cause for dementia, accounting for almost 70% of cases, and clinically characterized by memory loss, apathy and depression, impaired judgement, confusion, disorientation and other symptoms. Ageing is the main risk factor for AD. As there are only limited palliative therapeutic options for AD, the disease causes enormous personal and economic burden to society.

The pathological hallmarks of AD are extracellular plaques, composed of fibrillar amyloid-β (Aβ), and neurofibrillary tangles inside of neurons, composed of Tau, as already described by Alois Alzheimer in 1907 (Alzheimer et al. 1995).

Tau is a highly soluble and natively unfolded protein, mainly expressed in neurons, which is involved in the stabilization and organization of microtubules. Resulting from alternative splicing of 16 exons of the microtubule-associated protein Tau (MAPT) gene, located on chromosome 17q21, six isoforms are generated in the central nervous system (CNS). These can be divided into 4R Tau, containing 4 repeats (31–32 amino acids each), and 3R Tau, containing three repeats (lacking repeat 2; R2) (Wang and Mandelkow 2016).

The physiological functions of Tau are regulated by a variety of post-translational modifications, e.g. phosphorylation, glycation, acetylation, etc. In particular, the hyperphosphorylation of Tau is associated with its detachment from microtubules and pathological Tau aggregation (Morris et al. 2011).

Recent data have demonstrated that not only deposited fibrils (tangles) have toxic effects, but also that small, soluble protein oligomers play a fundamental role in AD pathology, as they cause synaptic and mitochondrial dysfunction. They might cause neurodegeneration a long time before protein fibrilization and deposition starts (Lasagna-Reeves et al. 2011; Kumar et al. 2014).

It has been shown that Tau assembly into paired helical filaments (PHFs) is strongly driven by two hexapeptide fragments within Tau: PHF6* (275-VQIINK-280) and PHF6 (306-VQIVYK-311). The PHF6 sequence is located at the beginning of the third repeat (R3) and can be found in all Tau isoforms. In contrast, the PHF6* sequence is placed at the beginning of the second repeat (R2) and is only present in four-repeat (4R) Tau isoforms. Both hexapeptide motifs show the highest predicted β-structure potential within the Tau sequence, and point mutations in the hexapeptide regions can change β-propensity, leading to an increase or decrease of aggregation (von Bergen et al. 2000, 2001; Barghorn et al. 2004).

The amyloid hypothesis states that in the progression of AD pathology, Aβ plaques appear first, leading to Tau hyperphosphorylation, tangle formation and neurodegeneration (Selkoe and Hardy 2016). However, the relationship and interplay of Aβ and Tau is still poorly understood. Recent data suggest that Tau pathology is not simply a downstream process of Aβ aggregation (Nisbet et al. 2015; Pourhamzeh et al. 2021).

Positron emission tomography (PET) imaging studies reveal that amyloid accumulation may predict the onset of Tau accumulation. However, Tau accumulation only predicts the onset of cognitive impairment, while the onset of Tau pathology occurs at the same time as the symptoms appear (Roe et al. 2013; Johnson et al. 2016; Hanseeuw et al. 2019). Pathological Tau aggregation, having a detrimental effect on neuronal function in preclinical models (Iqbal et al. 2005), spreads to various brain areas in a stereotypical pattern, correlating tightly with disease severity (Braak and Braak 1991). It has been demonstrated that Tau pathology spreads by intraneuronal transfer, a phenomenon denoted as “Tau pathology propagation”, in a prion-like way (Guo and Lee 2011; Holmes and Diamond 2014).

For these reasons, amongst others, Tau has come increasingly into focus for AD therapeutics research as an alternative or complement to Aβ-targeted therapeutic approaches (Lovestone and Manji 2020; Soeda and Takashima 2020). Several potential therapeutic substances targeting Aβ pathology, such as Aβ antibodies, secretase inhibitors or Aβ aggregation inhibitors, have failed in clinical trials due to numerous reasons. This however is similar to essentially all advanced clinical trials on AD (Cummings et al. 2018). Only the Aβ oligomer-targeting antibody Aducanumab has recently obtained a tentative FDA approval. However, this decision is still highly controversial, and the efficacy of Aducanumab has still to be proven in future clinical studies (Knopman et al. 2021; Mullard 2021; Walsh et al. 2021).

In order to reduce Tau pathology in AD, a variety of small molecules, including modulators of post-translational modifications and aggregation inhibitors of Tau, have been described. Most of them are in the preclinical stage (Bulic et al. 2013). Fourteen molecules have already entered clinical phases, but only the compound LMTM, a derivative of methylene blue, is currently under clinical investigation in phase III (Wang et al. 2021). According to the Alzforum webpage, earlier clinical trials with methylene blue derivatives failed (https://www.alzforum.org/therapeutics/lmtm).

Peptides, composed of two or more (up to 100) amino acids, represent a unique class of pharmaceutical compounds and are reasonable alternatives to chemical substances. Physiologically, they act as key regulators of biological functions and are attributed with high biological activity, valuable specificity, and in most cases, low toxicity (Lien and Lowman 2003; Danho et al. 2009). The preparation of peptides is simple, controllable and the peptides can be easily modified (Liu et al. 2016). They are often developed as drug candidates to interrupt protein–protein interactions. Currently, more than 400 peptide drugs are under clinical investigation, 60 are already approved in the USA, Europe or Japan (Lee et al. 2019).

Peptide drugs might also have disadvantages such as biological instability and membrane and blood–brain barrier impermeability (Henninot et al. 2018), but at least some peptides were demonstrated to cross cell membranes or/and the blood–brain barrier (Pappenheimer et al. 1997; Funke et al. 2010; Dammers et al. 2016; Zhang et al. 2020; Malhis et al. 2021; Aillaud et al. 2022). The problem of in vivo instability due to proteases can be overcome by chemical modification or the usage of D-amino acid peptides (Liu et al. 2010; Leithold et al. 2016; Lee et al. 2019). D-amino acid peptides have already been shown to be protease resistant and less immunogenic than the respective L-peptides (Schumacher et al. 1996; Chalifour et al. 2003; Sadowski et al. 2004).

**Fig. 1:**
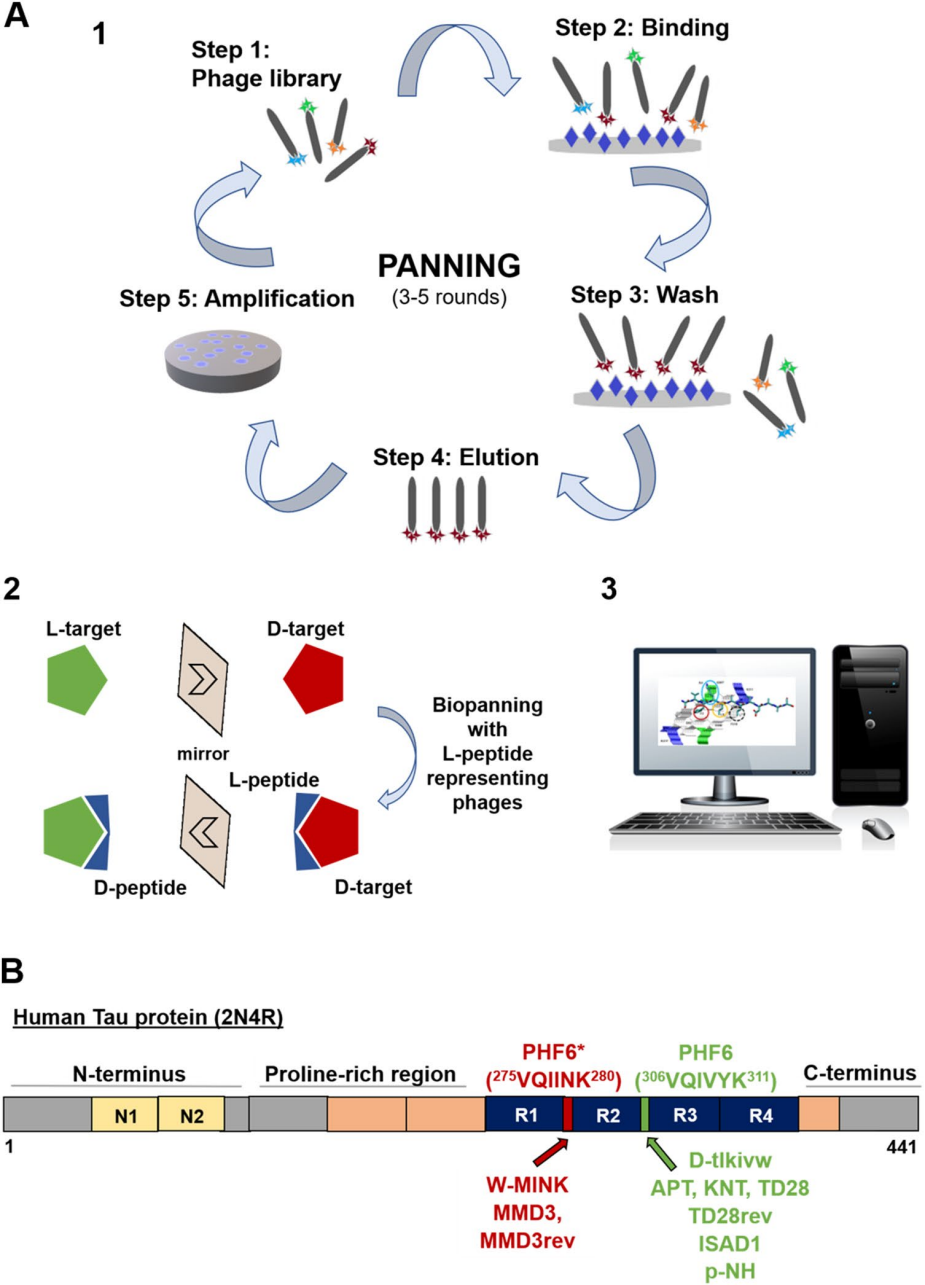
(D)-Peptide generation for application as Tau aggregation inhibitors (TAI). **A** Methods for identification of TAI peptides. (1) Phage display procedure. Target molecule immobilized on solid phase is incubated with a phage display library. Specific library phages bind to the target molecule and unbound phages are removed by washing. Bound library phages are eluted and then amplified in *E. coli*. Finally, the amplified phages are used in the next biopanning round. After serval rounds, phage DNA can be analyzed to obtain therapeutic peptides. (2) Principle of mirror image phage display. Target molecule is used as D-enantiomer in the selection process. Biopanning is performed with phages presenting L-peptides. Finally, the D-enantiomeric form of the selected L-peptide is synthesized. (3) Another method of peptide identification offers in silico modelling using a variety of software. **B** Binding sites of established peptides summarized in this article

Currently, a few peptide compounds developed as TAI have been described in the literature. All of them are still in preclinical stages, but one compound has already been successfully tested in AD mouse models. Here, we review the current state of research in this interesting and promising field of AD therapy development. The main results of current studies are summarized in Table 1. See Fig. 1 as a summary on (D)-Peptide generation for application as TAI.

**Table 1:** Overview of the main results of eight studies on the development of peptidic TAI

Name	Sequence	Generation	Results	Reference
TLKIVW	D-tlkivw	Computer-aided, structure-based design on Tau segment PHF6	Blocked fibril growth of PHF6, K12, K19 in vitro*.* IC_50_ values for Tau^FL^ fibrils 52.2 µM(Seidler et al. 2018)	(Sievers et al. 2011)
W-MINK	DVWMINKKRK	Computer-aided, structure-based design on Tau segment VQIINK (PHF6*)	Blocked Tau^FL^ fibril growth in vitro, reduced ability of exogenous Tau^FL^ fibrils to seed intracellular Tau in HEK293 biosensor cells. IC_50_ value of 1.1 µM for Tau^FL^	(Seidler et al. 2018)
D1; D1b; D1d	D-lyiwvq; D-lyiwiwrt; D-lyiwiqkt	Computer-aided, structure-based design on Aβ_16-22_	Peptides blocked Tau fibril formation in vitro and reduced ability of exogenous Tau^FL^ fibrils to seed intracellular Tau in HEK293 biosensor cells. IC_50_ value of 1.1 µM for Tau^FL^ for D1b. D1b also inhibited seeding of disease-relevant Tau derived from patient brain tissue	(Griner et al. 2019)
APT; KNT; TD28; TD28rev	D-aptllrlhslga; D-kntpqhrklrls; D-ttslqmrlyypp; D-ppyylrmqlstt	Mirror image phage display on PHF6-fibrils	Inhibit Tau fibril formation in vitro and in cell culture*.* IC_50_ value for 3RD-Tau between 6 and 200 µM. Penetrate neuronal cells, might have cytotoxic effects	(Dammers et al. 2016; Malhis et al. 2021)
MMD3; MMD3rev	D-dplkarhtswy; D-ywsthraklpd	Mirror image phage display on PHF6*fibrils	Inhibit Tau fibril formation in vitro*,* Tau is diverted into amorphous aggregates. IC_50_ values for Tau^RDΔK^ about 5 µM. Penetrate neuronal cells	(Malhis et al. 2021)
ISAD1; ISAD1rev	D- svfklsltdaas; D-saadtlslkfvs	Phage display on Tau^FL^	ISAD1 might bind to PHF6, inhibits aggregation of Tau^FL^ and disease-associated variants in vitro. IC_50_ values for Tau^RDΔK^ about 3 µM. Formation of non-toxic, non-fibrillar aggregates is favoured. Non-toxic to cells, inhibit toxic effects of Tau in cell culture	(Aillaud et al. 2022)
p-NH	D-nitmnsrrrrnh		Inhibits PHF6 fibril formation in vitro, reduces Tau phosphorylation and aggregation in cell culture, reduced NFT formation and improved cognitive abilities of Tau^P301S^ transgenic mice after intranasal application	(Zhang et al. 2020)
(TAT)-7H	YGRKKRRQRRR-HHHHHHH		TAT-7H suppressed Tau phosphorylation in iPS cell-derived neurons	(Kondo et al. 2021)

## Methods: Search Strategy and Selection Criteria

We have scanned novel publications listed on PubMed weekly since 2010 using the search terms “Alzheimer disease” and “Tau”. In addition, we performed searches using the terms “Tau”, aggregation inhibitors” and “peptides” and have analysed review articles on Tau aggregation inhibitors in general. All publications we have found reporting on peptides as Tau aggregation inhibitors to develop therapeutics for AD were included in this review article.

## Literature Overview

### Peptides Selected by Structure-Based Rational Design

The first study on Tau aggregation inhibiting peptides developed by computer-aided, structure-based design was published in 2011 by the group of David Eisenberg. The authors used a known crystal structure of the dual β-sheet “steric zipper” Tau segment VQIVYK (PHF6 sequence, located in R3 of Tau) as a template to design peptide aggregation inhibitors (Sievers et al. 2011). The “Rosetta” software (Kuhlman et al. 2003) was used to find non-natural peptides to target and block the ends of the PHF6 fibrils.

A tight interface was designed between the peptide and the fibril end to hinder addition of new building blocks. Four D-amino acid peptides were found, of which one, D-tlkivw, inhibited fibril formation of the PHF6-segment, but also of K12 and K19, two Tau constructs which lack the repeat R2 (Friedhoff et al. 1998), as shown by Thioflavin S (ThS) assays and electron microscopy (EM). Scrambled versions and the diastereomer, L-TLKIVW, were significantly less effective in inhibition of fibril formation. The position of the D-amino acid inhibitor at the end of fibrils of Tau K19 could be visualized using EM. Evaluation of nuclear magnetic resonance (NMR) spectroscopy data suggested a binding of D-tlkivw to PHF6 fibrils with an apparent dissociation constant of 2 µM, while the D-peptide seemed not to interact with monomers (Sievers et al. 2011).

However, whether the D-peptide inhibits full-length Tau (Tau^FL^) aggregation was not demonstrated in this article, as only shortened K12 and K19 Tau constructs missing R2 with enhanced aggregation propensity were used. Therefore, the very similar PHF6* sequence is missing in both constructs. Later, the group reported that D-tlkivw was not able to inhibit Tau^FL^ aggregation, suggesting that TAI compounds based on the PHF6 sequence might be less effective in aggregation inhibition of Tau^FL^ (Seidler et al. 2018).

In 2018, the same group reported on structures of fibrils of a VQIINK (PHF6*) containing segment, forming steric zippers, as determined by the cryo EM method micro electron diffraction (MicroED) (Seidler et al. 2018). Those structures were the starting point for the design of PHF6* inhibitor peptides that block Tau^FL^ aggregation and seeding. Bulky amino acid sidechains were modelled into the structure and amino acids were identified that could interfere with the two possible interfaces observed.

Two inhibitors derived from the PHF6* sequence, MINK and WINK, were found to inhibit Tau^FL^ fibril formation in Thioflavin T (ThT) assays. However, while WINK showed no detectable self-aggregation, MINK was found to self-aggregate to a certain extent. Both inhibitors could reduce the ability of exogenous Tau^FL^ fibrils to seed intracellular Tau in HEK293 biosensor cells expressing Tau-K18-(P301S)-EYFP in a dose-dependent way, whereas the VQIVYK (PHF6) targeted inhibitor D-tlkivw inhibited seeding poorly (IC_50_ value of 52.2 µM, compared to 22.6 and 28.9 µM for MINK and WINK, respectively).

A variety of possible phase 2 inhibitors were designed on the basis of a second structural polymorph of PHF6* amyloid fibrils, revealing another possible interface. MINK was redesigned to incorporate another steric clash to disrupt the third interface. The most effective inhibitor peptide, W-MINK, blocked Tau^FL^ fibril seeding even more successfully than MINK, with IC_50_ = 1.1 µM. Together, the results indicate that VQIINK (PHF6*) is, in comparison to PHF6, a superior target for TAI (Seidler et al. 2018). In this study, however, only one single PHF6 targeting peptide, developed in their own group, D-tlkivw, was used for comparison.

In 2019, the group of Eisenberg reported evidence supporting the hypothesis that Aβ-induced cross-seeding of Tau could promote tangle formation in AD. The idea was then to find peptide inhibitors able to prevent Aβ aggregation and to block the binding site of Aβ with Tau (Griner et al. 2019), presumably PHF6 and PHF6*, as already hypothesized (Guo et al. 2006; Miller et al. 2011). The authors used microED to determine the atomic structure of an Aβ steric zipper fibril-like fragment, Aβ_16-26_, containing the hereditary mutation D23N.

A Rosetta-based strategy was used to design capping peptide inhibitors on a search model of Aβ_16-22._ After the first round of design, four L-peptides and two D-peptides were selected for further characterization. None of the inhibitors was toxic to cells as shown in MTT-tests (3-(4,5-dimethylthiazol-2-yl)-2,5-didiphenyltetrazolium bromide), but only one inhibitor, designated D1, eliminated toxic effects of Aβ_1-42_ on Neuro-2a (N2a) cells at a 10 molar excess.

In a second round of design, six new inhibitors, designated D1a-D1f, were selected and tested. Two of them, D1b and D1d, reduced Aβ_1-42_ toxicity on N2a cells efficiently in a tenfold excess and equimolar ratio. D1, D1b and D1d elicited a dose-dependent response, and the IC_50_ value was estimated to be less than 1 µM. D1b and D1d were more effective than D1 in reducing fibril formation. The L-amino acid form of D1 did not reduce cell toxicity, as judged by MTT assay. The reduction of Aβ toxicity could be explained by a dose-dependent inhibition of Aβ aggregation, as demonstrated by ThT assays and transmission electron microscope (TEM) analysis. Antibody studies indicated that the inhibitors reduced Aβ oligomers as well as Aβ fibrils. In addition, the reduction of pre-formed Aβ aggregate toxicity by inhibitors D1b and D1d could be demonstrated with D1d being more effective, and there was evidence that pre-formed fibrils were either capped or coated.

Next, the authors could show that Aβ aggregates were able to seed Tau aggregation in Tau biosensor cells expressing Tau-K18 (P301S) EYFP, suggesting that there is an interaction between Tau and Aβ in the microtubule-binding domain. The inhibitor peptides were able to reduce seeding of Tau by aggregated Aβ, D1b being most effective. In addition, D1, D1b and D1d were able to reduce aggregation of Tau monomers, as demonstrated by ThT assays. They were not general amyloid inhibitors, as aggregation of human islet amyloid polypeptide (IAPP) and α-Synuclein was not inhibited. The inhibitors also prevented Tau^FL^ fibril seeding in Tau biosensor cells, D1b being the most effective inhibitor (IC_50_ value of 4.5 µM, 75 µM for D1d). Tau mutant experiments in Tau biosensor cells lead to the conclusion that D1b acted on the PHF6 and PHF6* segments of Tau. The authors hypothesized that PHF6 and PHF6* might share common structural features with the Aβ core. They could also demonstrate that seeding in Tau biosensor cells by amyloid species in brain-derived tissue from patients with AD or with progressive nuclear palsy could be prevented by D1b (Griner et al. 2019).

### Peptides Selected by (Mirror Image) Phage Display or Other

In 2016, the Funke group published the first of a series of articles describing the selection of D-amino acid peptides against Tau or peptides thereof. Phage display selections were performed using fibrils of the D-amino acid hexapeptide VQIVYK (PHF6). The selected D-amino acid peptides bound to PHF6 fibrils, Tau isoform fibrils such as 3RD-Tau (K19), as well as to Tau^FL^ fibrils, and modulated the aggregation of the respective Tau form, as demonstrated by ThT-tests and dynamic light scattering (DLS) (Dammers et al. 2016). In silico modelling suggested a binding motif similar to that of the tlkivw-D-peptide, developed by the Eisenberg group to bind PHF6 (Sievers et al. 2011). The D-peptides described by Dammers et al. were able to penetrate cells and slightly reduced the number of ThS-positive cells in an inducible N2aTau^RDΔK280^ cell culture model. The studies on the D-peptides were not pursued further because of D-peptide-mediated cytotoxicity (Dammers et al. 2016).

The group then employed another mirror-image phage display procedure to identify PHF6* fibril binding D-peptides. The identified D-amino acid peptide MMD3 and its inverse version, designated MMD3rev, inhibited fibrillization of the PHF6* hexapeptide, the repeat domain of Tau as well as Tau^FL^ in vitro, as demonstrated by Thioflavin assays. DLS, pelleting assays and atomic force microscopy (AFM) demonstrated that MMD3 prevented the formation of Tau fibrils rich in β-sheets by an interesting mechanism, partitioning Tau into large amorphous aggregates. Enzyme-Linked Immunosorbent Assay (ELISA) data demonstrated binding of MMD3 and MMD3rev to PHF6*- and Tau^FL^ fibrils, while NMR measurements suggested that they bound to monomeric Tau^FL^ only with rather low affinity (Malhis et al. 2021). The binding mode to PHF6* fibrils resembled the binding mode of the W-MINK peptide, developed by the Eisenberg group to block PHF6* fibrils (Seidler et al. 2018). The PHF6* targeting peptides MMD3 and MMD3rev identified were able to penetrate neuronal cells (Malhis et al. 2021).

Recently, the group aimed at the generation of D-peptides which bind to Tau^FL^. Tau^FL^ binding D-peptides are of great therapeutic interest because they can potentially inhibit aggregation of the Tau protein at the beginning of the fibrillation cascade. The D-peptides binding to Tau^FL^ could stabilize the non-toxic and physiologic form and prevent the oligomerization process in the earliest phases of the fibrillization pathway. First, a Tau^FL^ binding L-peptide, ISAL1, was selected and synthesized as D- and retro-inverse form (ISAD1 and ISAD1rev). The D-peptides were characterized with respect to their specificity for Tau conformers (monomers and aggregates) and their therapeutic potential. Using ELISA and fluorescein amidite (FAM)-labelled peptides, binding of the peptides to both Tau^FL^ and Tau^FL^ fibrils was demonstrated. The aggregation-prone hexapeptide motif within Tau, PHF6, was identified as a possible binding site of the most promising D-peptide ISAD1. ISAD1 inhibited fibrillization of Tau^FL^ and a wide variety of disease-relevant Tau isoforms (Tau^RDΔK280^, Tau^FLΔK280^, Tau^FL−A152T^, Tau^FL−P301L^). Similar to the D-peptide binding PHF6* described above, it was found that the D-peptides reduced regular Tau fibril formation by forming large non-fibrillar, non-toxic aggregates, which were ThT negative. They were examined in more detail with respect to their particle size in DLS, pelleting assay and western blot. ISAD1 and ISAD1rev were tested in cell culture, where it was evident that the peptides were taken up by neuronal Tau expressing cells and accumulated in the cytosol. The peptides were non-toxic to cells and prevented Tau fibril-mediated cell toxicity of externally added and internally expressed Tau (Aillaud et al. 2022).

In 2020, Zhang and colleagues reported a study similar to that of Dammers et al., 2016. Mirror image phage display was performed on PHF6 fibrils to obtain D-peptides inhibiting the formation of PHF6 fibrils, as demonstrated by ThT assays and EM. The D-peptide p-NH (nitmnsrrrrnh) was able to enter neuronal cells and inhibited Tau hyperphosphorylation and fibrillization. After intranasal application, it improved the cognitive abilities of Tau^P301S^ transgenic mice, reducing NFT formation (Zhang et al. 2020).

In 2021, Kondo and colleagues reported on hepta-histidines (7H), which unexpectedly inhibited R3-Tau aggregation in a dose-dependent way, as shown by DLS and EM. 7H was originally investigated as an inhibitor on Ku70 and Huntingtin protein interaction. The peptide transiently contacted Tau at multiple sites with a possible preference for PHF6*. Addition of the trans-activator of transcription (TAT)-sequence to 7H increased its cell permeability. In human neurons differentiated from homozygous Tau^P301S^-induced pluripotent stem (iPS)-cells, (TAT)-7H inhibited Tau phosphorylation at Ser202 and Thr205 (Kondo et al. 2021).

## Discussion

Peptides, especially D-amino acid peptides, can be interesting alternatives to small chemical molecules or antibodies. Cell and blood–brain barrier permeability are possible advantages of at least some peptides over therapeutic antibodies, which might have a higher affinity towards their target molecules but cross membranes only poorly (Matsson et al. 2016). Compared to small chemical molecules, the larger binding surface of therapeutic peptides might be of advantage, promising a more successful inhibition of protein–protein interactions (Petta et al. 2016). All of the peptides reviewed here have IC50 values between 1 and 50 µM. The question of whether further optimization of the peptides therapeutic properties might be necessary, will need to be evaluated in animal studies.

As peptides are produced synthetically, they can easily be modified and optimized to fulfil the demands of the pharmaceutical industry. Using methods such as alanine-scans, one can investigate which of the amino acids are essential to perform the desired biological functions (Morrison and Weiss 2001). Amino acid scanning techniques, including pepspot membranes, or molecular modelling approaches allow optimization with respect to e.g. peptide affinity (Funke and Willbold 2012; Eustache et al. 2016; Klein et al. 2016).

Recently, it was demonstrated that therapeutic D-amino acid peptides can be absorbed systematically after oral or intranasal administration (Pappenheimer et al. 1997; Funke et al. 2010; Zhang et al. 2020). The Aβ-binding D-peptide D3 (amino acid sequence rprtrlhthrnr) reduced plaque load and cerebral inflammation of AD mouse models after oral treatment, and the cognitive performance was significantly increased if compared to untreated control mice (Funke et al. 2010). D3-derivatives demonstrate excellent blood–brain barrier permeability and were successfully optimized with respect to their therapeutic potential (Jiang et al. 2015; Klein et al. 2016; Leithold et al. 2016). The D3-derivative RD2 recently passed a first clinical study in men (Kutzsche et al. 2020), clinical studies of phase two are planned.

The D3 and RD2 sequences contain arginines, so do the sequences of the D-peptidic TAI p-NH described by Zhang and colleagues (Zhang et al. 2020) and the (TAT)-7H TAI peptide described by Kondo and colleagues (Kondo et al. 2021). Already in 2017, Nadimidla and colleagues found that poly-L arginine hydrochloride inhibited aggregation of PHF6 and a PHF6* containing fragment as well as of Tau mutant protein P301L (Nadimidla et al. 2017). The impact of arginines and arginine-rich peptides is very interesting and was reviewed by Mamsa and Meloni in 2021 in detail (Mamsa and Meloni 2021).

Most of the peptides described as TAI target the PHF6 or the PHF6* sequence motifs of Tau. It remains to be seen which of the sites is the more potent driver for Tau aggregation and therefore the more interesting target for TAI. Seidler and colleagues suggested that PHF6* is the most promising target for the development of TAI (Seidler et al. 2018). In our group, we did not find serious differences in preliminary in vitro studies using D-amino acid peptides selected against PHF6 or PHF6* (Malhis et al. 2021). It is known that in vivo PHF6* within R2 is present in AD patients only in 50% of neuronal Tau (3R vs. 4R Tauopathies, see (Goedert et al. 1989)). Recent cryo-EM studies even demonstrated that Tau fibrils extracted from AD brains have a core composed of R3, R4 and ten residues beyond the end of R4 (Fitzpatrick et al. 2017), suggesting that PHF6 might be the most valuable target for development of TAI. On the other hand, Seidler et al. hypothesized that the core of the fibrils was not the primary driver of aggregation, but might serve as a solvent-excluded scaffold that can cluster PHF6* together in the fuzzy coat, which poises the solvent-exposed VQIINK steric zippers for seeding (Seidler et al. 2018).

Fitzpatrick and colleagues have also shown that Tau filaments extracted from patients with different tauopathies differ in structure (Fitzpatrick et al. 2017). The implications of this and the different TAI described in this review have not yet been tested. Only in vivo studies can answer the question which of the peptides presented in this review will be the most promising leads for AD therapy research. Until now, successful animal studies have only been performed for p-NH as described by Zhang and colleagues (Zhang et al. 2020).

## Conclusion

In recent years, a variety of peptide compounds acting as Tau aggregation inhibitors were developed as a promising therapeutic approach towards Alzheimer disease. In particular. the suitability of D-amino acid peptides for possible in vivo applications has already been demonstrated. Most of the peptides already described in the literature were developed by structure-based design or phage display selections. The therapeutic properties of those peptides were summarized in this manuscript. Molecular insight into the binding mode of different peptides was gained by in silico modelling. Some of the identified Tau-targeting peptides were able to penetrate neuronal cells, reduce Tau phosphorylation or improved cognitive abilities of Tau transgenic mice, making them interesting for AD therapy. Future studies are needed to demonstrate which of the peptides can be developed into a therapeutic compound for the treatment of AD.

## Data Availability

Not applicable.

## Abbreviations

7H: Hepta-histidine
Aβ: Amyloid-β
AD: Alzheimer disease
AFM: Atomic force microscopy
AGD: Argyrophilic grain disease
APP: Amyloid-precursor-protein
CNS: Central nervous system
CTE: Chronic traumatic encephalopathy
D23N: Hereditary mutation in Aβ
DLS: Dynamic light scattering
ELISA: Enzyme-linked immunosorbent assay
EM: Electron microscopy
EYFP: Enhanced yellow fluorescent protein
FAM: Fluorescein amidite
FDA: Food and drug administration
hIAPP: Human islet amyloid-precursor-protein
HD: Huntington disease
iPS: Induced pluripotent stem cells
K18: Construct of 4R Tau
K19: Construct of 3R Tau
Ku70: DNA repair subunit protein
MAPT: Microtubule associated protein Tau
MicroED: Micro electron diffraction
MTT: 3-(4,5-Dimethylthiazol-2-yl)-2,5-didiphenyltetrazolium bromide
N2a: Neuronal-2a cell line
NFTs: Neurofibrillary tangles
NMR: Nuclear magnetic resonance
P301S: Missense mutation
PET: Positron emission tomography
PHFs: Paired helical filaments
PART: Primary age-related tauopathy
PS1: Presenilin-1
PSP: Progressive supranuclear palsy
R1-4: Microtubule binding repeats 1–4
RD: Repeat domain
TAI: Tau aggregation inhibitors
TAT: Trans-activator of transcription
Tau^FL^: Full-length human Tau (441 amino acids)
Tau^RDΔK^: Pro-aggregant repeat domain Tau mutant ΔK280
Tau^FLΔK^: Full-length Tau mutant ΔK280
Tau^FL^^−^^A152T^: Full-length Tau mutant A152T
Tau^FL^^−^^P301L^: Full-length Tau mutant P301L
TEM: Transmission electron microscopy
ThT: Thioflavin-T
ThS: Thioflavin-S
